# An open prospective study evaluating efficacy and safety of a new medical device for rectal application of activated carbon in the treatment of chronic, uncomplicated perianal fistulas

**DOI:** 10.1007/s00384-016-2726-z

**Published:** 2016-11-23

**Authors:** Antoni Zawadzki, Louis Banka Johnson, Måns Bohe, Claes Johansson, Mats Ekelund, Ole Haagen Nielsen

**Affiliations:** 1 Department of Clinical Sciences, Division of Surgery, Skåne University Hospital, Lund University, Malmö, Sweden; 2 Department of Surgery, Danderyd Hospital, Karolinska Institutet, Stockholm, Sweden; 3 Department of Gastroenterology D112, Medical Section, Herlev Hospital, University of Copenhagen, Herlev Ringvej 75, DK-2730 Herlev, Denmark

**Keywords:** AST-120, Activated carbon, Perianal fistula, Rectal instillation, Treatment

## Abstract

**Purpose:**

It has been proposed that biological/chemical substances in the intestine might play a role in the occurrence and deterioration of perianal fistulas. Elimination of such unidentified factors from the lower gastrointestinal tract might offer a new strategy for the management of anal fistulas. The aim of this study was to evaluate the clinical effects on non-Crohn’s disease perianal fistula healing, and the safety and tolerability of a new medical device that applies high-purity, high-activity granular activated carbon locally into the rectum twice daily of patients with perianal fistulas without any concomitant medication.

**Methods:**

An open, single-arm, prospective study with active treatment for 8 weeks and an optional follow-up until week 24 (ClinicalTrial.gov identifier NCT01462747) among patients with chronic, uncomplicated perianal fistulas scheduled for surgery was conducted.

**Results:**

Of 28 patients included, 10 patients (35.7%) showed complete fistula healing (closed, no discharge on palpation) after 8 weeks; seven of these patients, corresponding to 25% of the enrolled patients, remained in remission for up to 31 weeks. At week 8, there was a statistically significant reduction in the discharge visual analog scale (*p* = 0.04), a significant improvement in the patient-perceived quality of life for the category of embarrassment (*p* = 0.002), and a trend toward improvement in the other assessment categories.

**Conclusions:**

The treatment was well tolerated, and patient acceptance was high. The results support the efficacy and safety of locally administered activated carbon for the treatment of patients with chronic uncomplicated perianal fistulas not receiving any other medication for fistula problems.

## Introduction

Perianal fistulas are often bothersome to affected patients and may be considered to be a chronic disease because the spontaneous cure rate is low. Available treatment options are limited; usually, surgery is performed [1] with its associated risks. Thus, the consequences of anal surgery potentially include profound fecal incontinence and an impaired quality of life [2]. Hence, there is a dire need for new treatment options for this condition.

The etiology of perianal fistulas is believed to be cryptoglandular infection, often in individuals with a previous history of an anorectal abscess [3], although they may additionally arise due to more specific causes, e.g., Crohn’s disease (CD) [4], malignancy, or following radiation therapy [5]. The local microbial flora in these situations may facilitate an inflammatory cascade and initiate production of tissue-damaging chemical/biological substances in the perianal area (e.g., endotoxins, oxygen-reactive species, cytokines, and arachidonate metabolites). Such factors combined with other tissue-damaging fecal substances like bile acids and enzymes might lead to a local functional derangement with epithelial defects and the occurrence of perianal fistulas. Therefore, elimination of unidentified substances from the gastrointestinal tract might offer a novel therapeutic strategy.

Activated carbon is a well-tolerated substance with widespread use owing to its outstanding nonspecific adsorptive properties and its use for detoxification, and there is no systemic absorption after oral or rectal administration. Previously orally administered activated carbon (AST-120) has been investigated on anal fistulas in CD, however, with conflicting results [6, 7], as its passage through the intestinal tract may possibly influence the adsorptive capacity. Thus, by using oral administration of activated carbon, it is uncertain how much adsorption power is actually available when it reaches the rectal ampulla.

However, by administration of activated carbon into the rectum, a much higher adsorption power might be available in the perianal region. This is the first study evaluating the effects of activated carbon applied directly at the site of action on the healing of non-CD perianal fistulas, by means of a new medical device (ASTER) using a rectal applicator.

## Materials and methods

### Study design

An open, uncontrolled, nonrandomized, single-arm prospective study (Kulist-001, ClinicalTrials.gov identifier NCT01462747) evaluating the efficacy and safety of a medical device administrating activated carbon for the treatment of chronic, uncomplicated, and non-CD perianal fistulas was performed. The clinical study was approved by the local scientific ethics committee and competent authority.

### Patient population

Patients were in the age group of 18–75 years with suspected perianal fistula complains requiring referrals to tertiary surgical centers. All patients underwent a clinical examination as well as a 3D ultrasound to confirm diagnosis of active trans-sphincteric fistulas according to Parks’ classification [8], and to exclude extra-suprasphincteric fistula activity or other diseases. Diagnosis of an active/open fistula was based on case history, clinical, and ultrasound examination. Only patients with active simple anal fistulas without any side tracts or cavities that were assessed and scheduled for surgery (i.e., placed on a waiting list) were given the option of being included into the study.

There were no restrictions on therapy received prior to this study. However, any anti-inflammatory therapy (NSAIDs), antibiotics, or concomitant immunomodulatory therapies (including glucocorticoids, thiopurines, and tumor necrosis factor-α inhibitors), as well as any other therapy against fistula(s), were not allowed during the trial.

### Study visits and medical device

Four study data points were scheduled for patients included into the trial: day 1, baseline (site visit); week 2 follow-up (telephone call); week 8 follow-up (site visit), and a final optional follow-up after a minimum of 24 weeks (site visit). Blood samples for measurement of C-reactive protein, hemoglobin, and white blood cell counts were collected at baseline and week 8.

**Fig. 1:**
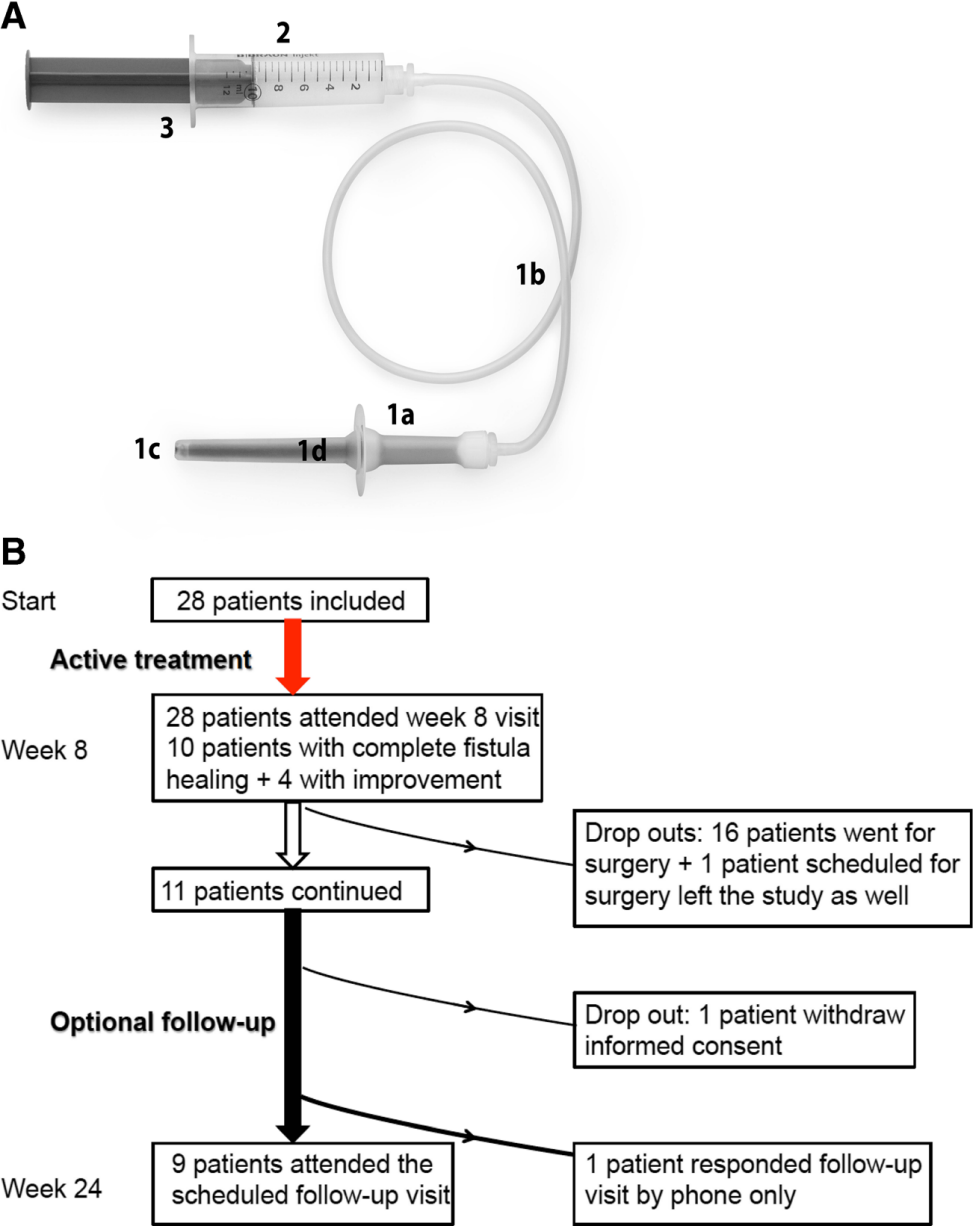
**a** The medical device assembled ready for use: *1a* Rectal cannula (with cap removed) and carbon chamber. *1b* Connection tube. *1c* Vaseline lubricated tip. *1d* Activated carbon (1.2 g). *2* Sterile water, 10 mL (EUP quality). *3* Sterile syringe (CE-marked), 10 mL. **b** Flowchart of patient disposition during the trial (active treatment and follow-up)

The high-purity, high-activity granular activated carbon was delivered rectally using the medical device (QPharma, Limhamn, Sweden). The rectal treatment was administered twice daily with a minimum of 6 h between administrations. The device (Fig. 1a, assembled device) is for single use only and consists of (1) a rectal applicator with a Vaseline-lubricated tip with a protective cap, a carbon chamber containing 1.2 g of activated carbon (EUP 2010), and a connector tube; (2) sterile water (EUP quality) 10 ml from a plastic container (Fresenius Kabi, Uppsala, Sweden); and (3) a 10-ml sterile syringe (Braun Medical, Bethlehem, PA, USA). The syringe is filled with sterile water and connected to the rectal applicator. When injected into the rectal applicator, the sterile water acts as a propellant to push out the activated carbon.

### Endpoints

The primary endpoint of the study was the clinical evaluation of fistula healing, healed/not healed (healed defined as closed, no discharge on palpation) at week 8. Secondary endpoints were included change (baseline to end of study) of perianal symptoms as assessed by the patient (global assessment of discharge and pain reported on a VAS scale), as well as impact on daily functions (patient-perceived quality of life), tolerability, and safety. Change in healing assessed by subject-reported subjective symptoms (discharge and pain on a published VAS scale previously used in this field [9]) were presented as percent change and absolute change from baseline. A validated patient assessment questionnaire earlier applied was used to evaluate patient-perceived quality of life using the following parameters: embarrassment, functionality, fear of surrounding, sexual activity, risk of spontaneous leaking, traveling and toilet localization [10].

### Statistical analysis

The sample size of a minimum of 25 patients was based on identical statistical assumptions as described in the Fukuda trial [7], with an estimated spontaneous remission rate of 10% and a target remission rate of 35% at week 8.

## Results

A flowchart showing the disposition of patients is provided in Fig. 1b. The mean age of patients was 46 years (22 were males (78.6%)). All 28 patients included were diagnosed with a trans-sphincteric fistula, and 9 of 28 patients (32.1%) had received nonsurgical treatment for current or previous fistula(s).

After 8 weeks of treatment, 10 of 28 patients (35.7%) showed complete clinical fistula healing. In addition to the 10 patients with clinical healing at week 8, four patients experienced improvements but no healing according to predefined criteria.

Eleven of the 28 patients (39.3%) continued in the optional 24-week follow-up study (Fig. 1b). Seventeen patients already placed on the waiting list, however, accepted surgery after week 8 (Fig. 1b). At week 24, data were available for 9 of 10 patients (one patient withdraw informed consent, cf. Fig. 1b) with clinical healing at week 8 (although an additional patient did not show up to the scheduled week 24 visit but contacted the ward by phone and reported to be without fistula symptoms). Thus, 7 of 9 patients attending the week 24 visit, corresponding to 25% of the original number of treated patients, had maintained remission at week 24 (or 8 of 10 patients (i.e., 29% of all patients treated) if including the patient who reported only by telephone).

A 26-point median reduction (*p* = 0.04) in discharge VAS was observed after 8 weeks, and a further median reduction of 6.5 points (i.e., 32.5 points from baseline; *p* = 0.005) was observed in patients attending the week 24 follow-up. However, no effect was observed on the pain VAS score as assessed after 8 and 24 weeks.

For the patient assessment questionnaire at week 8, significant improvements were observed from baseline for the category of embarrassment (*p* = 0.002). Improvements were noted for functionality, traveling, fear of surroundings, and toilet location. At week 24, there were significant improvements in embarrassment (*p* = 0.034), health conditions (*p* = 0.034), and risk of spontaneous leakage (*p* = 0.038).

Patient acceptance of the medical device was sound, and a mean compliance was 99%. Four patients experienced a total of seven adverse events during the study; all events were judged to be mild (e.g., nausea and constipation), and all were resolved by completion of the study without any treatment required. Further, no clinically significant abnormal laboratory results were reported during the study.

## Discussion

A majority of patients with clinical healing/remission at week 8 maintained remission at week 24. The recurrence rate, for obvious reasons, needs to be a primary endpoint [11]. Here, the recurrence rate at 24 weeks was 22% if only the patients with clinical healing at week 8 were considered, but if all patients were taken into account, the recurrence rate was 75%. In comparison, for fistula plug treatment, a recurrence rate of up to 76% has been reported [12].

Patients reported statistically significant improvements from baseline to week 8 for the category of embarrassment and improvements for functionality, traveling, fear of surroundings, and toilet location. At week 24, statistically significant improvements in embarrassment, health conditions, and risk of spontaneous leakage were observed.

In conclusion, a huge need for novel treatment options of chronic, perianal fistulas exists. Rectal application of high-purity, high-activity granular activated carbon with a new medical device might be a promising and safe alternative treatment option for patients with chronic uncomplicated perianal fistulas and may additionally preserve sphincter function. However, based on the data presented and the limitations of an open study, a larger randomized, controlled trial with a minimum of 12 months of follow-up is highly warranted.
